# Obesity and Overweight Risk Factors in Sudden Death Due to Cardiovascular Causes: A Case Series

**Published:** 2017-06

**Authors:** Dan PERJU-DUMBRAVA, Ovidiu CHIROBAN, Carmen Corina RADU

**Affiliations:** 1. Dept. of Legal Medicine, School of Medicine, UMF Iuliu Hatieganu University, Cluj-Napoca, Romania; 2. Dept. of Bioethics and Legal Medicine, School of Medicine, University of Oradea, Oradea, Romania

## Dear Editor-in-Chief

Obesity is most often secondary to an increased food intake in subjects with a certain genetic predisposition. However, it may result because of metabolic disorder, endocrine malady and brain tumor or traumatic lesion. It generates numerous cardiovascular complications, metabolic etc. and its primary treatment is dietary. There were about 20 million cardiovascular disease (CVD) deaths in 2015, accounting for 30% of all deaths worldwide (1, 2). Nowadays, from the non-violent deaths, sudden death due to CVD causes is the main death threat. Therefore, it represents a major problem of public health, although there are multiple alertness campaigns in cardiovascular medicine and emergency medical facilities are making considerable efforts, it persists as a major cause of mortality in industrialized countries, including Romania. All current efforts are directed towards medical sciences that analyze from the pathophysiological point of view and try to elucidate the underlying substrate of sudden cardiac death. The importance of understanding this phenomenon is based on the identification of structural, functional and triggering factors in order to establish which of the individuals in the general population are most likely to die suddenly, in this way, we can enforce targeted preventive measures, thus eradicate the risk factors, and avoid the triggering factors.

We conducted a study using death registers, death certificates and forensic reports belonging to Forensic Service Bihor County in the period 1 Jan 2014-31 Dec 2014. Overall, 96 persons passed away due to sudden cardiac death recorded in the studied period. Regarding obesity, this risk factor analysis was determined by the body mass index of all members of the study group in determining the thickness of the adipose tissue measured in the abdominal wall. Following the autopsy of all the 96 cases of suspicious death, of which 20 females and 76 males, in order to elucidate the cause, the results were: acute myocardial infarction was indicated as cause of death in 53% of cases. The second cause, featured in 26% of cases, was myocardial sclerosis, with 25 cases; followed by cardiomyopathies (dilated, hypertrophic and mixed), totaling 13 people; it followed: myocardial rupture in 1.04% of cases-one female case, myocarditis 2%, 2 cases, and high blood pressure occurring in 4% of the persons-4 deaths.

The data from the analysis results of the deceased persons by sudden cardiac death in Bihor County in Jan 2014 to Dec 2014 correspond to the data existing in literature. As it is already well known, myocardial infarction is the leading cause of death in industrialized countries. Risk factors for coronary heart disease mix with those of sudden death.

An important aspect that I would like to emphasize from the details of my research is that sudden cardiac death, with the pathological substrate of acute myocardial infarct, is seen in people relatively young, especially among males who registered the maximum number of deaths between 51–60 yr. The second largest cause of death was caused by myocardial sclerosis, which does not explain by itself the cause of death because it is a chronic lesion fund requiring a contributing factor to trigger the death-resulting chain. Myocardial rupture is a complication of AMI that directly causes death in 8% of patients (3).

We note that obesity, consisting of well and very well represented muscle-adipose tissue is known as a risk factor for cardiovascular disease mortality and sudden cardiac death. The distribution of abdominal adiposity is also known as a risk factor, which along with high serum levels of triglycerides, low levels of HDL cholesterol, diabetes mellitus type 2 and the high blood pressure are part of the metabolic syndrome (4). In addition, the newest researches show that high serum levels of free fatty acids are incriminated as factors with a role in triggering cardiac arrhythmias (5, 6). The importance of this article consists of the fact that it proves once again that one of the main “killers” of the modern world is preventable. Although in theory, obesity is definitely a risk factor for cardiovascular deaths, organizing and compiling data regarding a massive number of premature deaths can give one an unbeatable sense of self-consciousness regarding this very important public health problem. Measures should be taken in order to prevent the apparition of obesity, which includes a healthier lifestyle with a balanced diet and proper physical exercise.
